# Proliferative Vitreoretinopathy: A Reappraisal

**DOI:** 10.3390/jcm12165287

**Published:** 2023-08-14

**Authors:** Paolo Carpineto, Arturo Maria Licata, Marco Ciancaglini

**Affiliations:** 1 Department of Medical, Oral and Biotechnological Sciences, University “G. d’Annunzio” of Chieti-Pescara, 66100 Chieti, Italy; paolo.carpineto@unich.it; 2 Department of Clinical Medicine, Public Health, Life and Environmental Sciences, University of L’Aquila, 67100 L’Aquila, Italy; marco.ciancaglini@cc.univaq.it

**Keywords:** retinal detachment, proliferative vitreoretinopathy, vitreoretinal surgery, epithelial mesenchymal transition, subretinal fluid

## Abstract

Proliferative vitreoretinopathy (PVR) remains the main cause of failure after retinal detachment (RD) surgery. Despite the development of modern technologies and sophisticated techniques for the management of RD, the growth of fibrocellular membranes within the vitreous cavity and on both sides of the retinal surface, as well as intraretinal fibrosis, can compromise surgical outcomes. Since 1983, when the term PVR was coined by the Retina Society, a lot of knowledge has been obtained about the physiopathology and risk factors of PVR, but, despite the proposal of a lot of therapeutic challenges, surgical skills seem to be the only effective way to manage PVR complications.

## 1. Introduction

Since 1920, when Gonin first described a successful retinal detachment (RD) surgical treatment [1], RD surgery has made extraordinary progress and achieved significantly improved success rates thanks to the development of several technologies and increasingly sophisticated techniques. However, about 10% of patients currently require additional surgeries to repair recurrent RDs, and the main cause of surgical failure is proliferative vitreoretinopathy (PVR) development.

**Figure 1:**
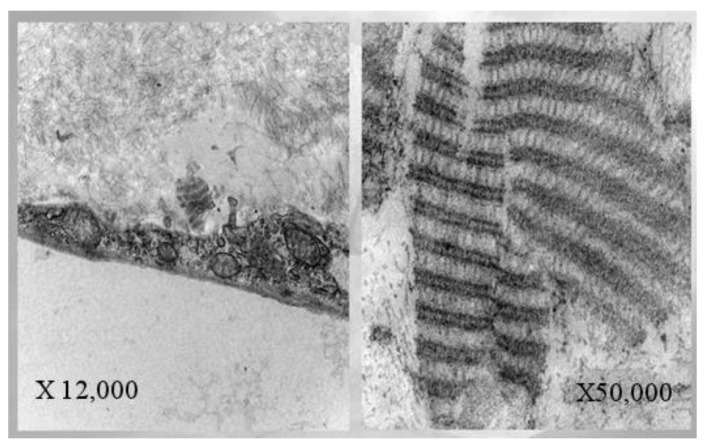
Histological section of subretinal PVR membrane shows long-spaced collagen (type IV) on different magnifications.

The term PVR was introduced in 1983 by the Retina Society Terminology Committee [2] and refers to an abnormal scarring process that complicates RD [3]. It is characterized by the growth of fibrocellular membranes within the vitreous cavity and on both sides of the retinal surface, as well as intraretinal fibrosis. Due to the presence of myofibrils, these epi-/subretinal membranes can contract, inducing tractional RDs with fixed retinal folds, even due to the contents of collagen in the extracellular matrix (seen in Figure 1).

## 2. Epidemiology and Risk Factors

The incidence of PVR has remained substantially unchanged over the last 25 years, despite the evolution of vitreoretinal surgical techniques [4]. PVR tends to complicate from 5.1 to 11.7% of rhegmatogenous RDs [3]. The incidence of recurrent retinal detachment due to PVR is higher within the first three months after surgery, with 77% of cases occurring within one month and 95% of cases within 45 days. In the presence of PVR, RD surgery has an anatomical success rate of 45–85%, while the functional success rate (defined as a final visual acuity of 5/200 or higher) ranges between 26% and 67% [5].

Only 10 to 40% of retinal detachments with PVR result in anatomic success, despite repeated surgery attempts [6,7]. In addition, only 40 to 80% of patients who achieve anatomic success achieve so-called “ambulatory vision”, meaning vision that is good enough to see large objects at close range [6].

The risk factors for PVR development have been classified as preoperative, intraoperative, and postoperative, as well as behavioral and genetic.

The preoperative risk factors include a large extent of RD, retinal breaks greater than one clock hour, multiple or undetected retinal breaks, vitreous hemorrhage, choroidal detachment, aphakia, previous failed attempts in reattachment, prolonged intraocular inflammation, high myopia, previous retinitis, and traumatic open-globe injuries [8].

The intraoperative risk factors include vitreous or subretinal hemorrhage, excessive cryotherapy or photocoagulation, loss of vitreous and retinal incarceration during subretinal fluid (SRF) drainage, and the incomplete sealing of retinal tears. 

The postoperative risk factors include uveitis, intraocular hemorrhage after surgery, choroidal detachment, multiple surgical procedures, persistent traction on retinal tears, and air tamponade [5,7,8].

The main behavioral risk factor identified in PVR development was cigarette smoking after both traumatic [9] and primary retinal detachment [10].

Some variants of inflammatory mediators and cell cycle regulators have been considered genetic risk factors. Polymorphisms in TNF and IL1A correlated with postoperative PVR [11,12]. Another link has been found between PVR after primary RD repair and a T309G polymorphism in the *MDM2* gene [13]. In addition, in a separate case–control study, a *p53* gene polymorphism affecting protein function was associated with a greater risk of postoperative PVR in subjects undergoing surgery for primary RD [14]. Furthermore, polymorphisms in the apoptosis mediators BAX and BCL-2 have been identified as predictors of PVR risk [15].

## 3. Physiopathology: “EPR Dysfunction”

PVR is a multifactorial process whose trigger is the RD itself, since it implies two important phenomena: the blood–retina barrier breakdown and the retinal hypoxia resulting in photoreceptor death. The alteration of the interface between neuroretina and retinal pigment epithelium (RPE) causes the activation of RPE and glial cells that migrate into the vitreous cavity and onto the retinal surface [6,16].

RPE cell transdifferentiation plays a critical role in several retinal diseases, including hereditary retinal dystrophies and age-related macular degeneration, and it represents a key event in the pathogenesis of PVR [17]. The RPE consists of a single layer of highly polarized cells lying between photoreceptors and choriocapillaris. There are approximately 3.5 × 10^6^ RPE cells in each adult human eye. The proper functioning of RPE requires a specific polarized distribution of transmembrane proteins. For example, Na^+^/K^+^-ATPase, intracellular chloride channel, mannose receptors, and monocarboxylic transporter (MCT)1 are confined to the apical portion of RPE cells, while integrins, MCT3, and Bestrophin-1 are located on the basal membrane. In addition, RPE cells also secrete proteins in a polarized manner [18,19,20]. The vascular endothelial growth factor (VEGF) is secreted primarily in the basal direction to promote the growth of the vascular choroidal system, while the pigment epithelium-derived factor (PEDF), an angiogenic inhibitor, is secreted apically [21,22].

During embryogenesis, the cells’ ability to switch between the epithelial and the mesenchymal statuses is crucial for the development of the human body [21]. These processes are known as Epithelial/Mesenchymal Transition (EMT) and Mesenchymal/Epithelial transition (MET). In healthy tissues, fully differentiated epithelial cells typically perform specific functions and they are not able to transdifferentiate. However, EMT can be activated in pathological circumstances, such as inflammation, wound healing, and carcinogenesis, allowing epithelial cells to achieve a greater migration capacity and to increase their production of extracellular matrix components [23].

Historically, EMT is classified into three subtypes: type I EMT occurs in the early stages of embryogenesis; type II EMT is associated with tissue regeneration and organ fibrosis; type III occurs in cancerous cells and is responsible for invasion and metastasis [24].

The loss of epithelial markers, including zona-occludens-1 (ZO-1), E-cadherin, and cytokeratin, and the gain of mesenchymal markers, including vimentin, N-cadherin, and fibronectin, are typically used to identify EMT. Recently, CD44 and the transmembrane protein Podoplanin have been pointed out as potential key actors in EMT [25]. 

However, the definition of EMT remains strongly debated because it is considered oversimplified. In fact, the gain of the ability to invade and migrate is not necessarily accompanied by the complete loss of epithelial traits. It has been proposed that EMT and MET represent the extremes of a continuum, with the existence of intermediate phenotypes. 

Adult RPE cells retain the ability to move along a continuum between polarized epithelial cells and mesenchymal cells. The concept of epithelial dysfunction has recently been proposed to define the transition from fully differentiated epithelial cells to mesenchymal cells (including intermediate statuses) [26].

**Figure 2:**
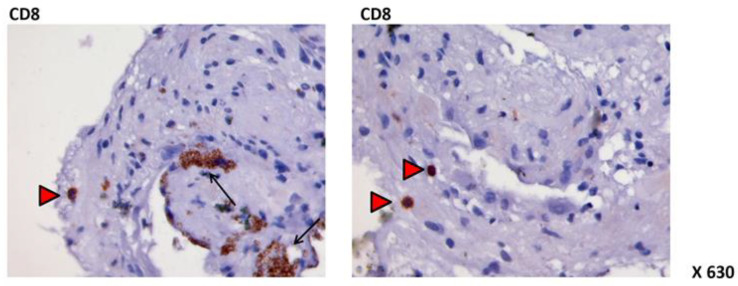
Immunohistochemistry on subretinal PVR membrane. Red arrows show presence of CD8 T-cells. Black arrows show nonspecific positivity to other cell markers.

Multiple inflammatory cells and mediators have been identified in the vitreous humor and within the epiretinal membranes of eyes with PVR, including macrophages, CD4+ and CD8+ T-lymphocytes, and B-lymphocytes (shown in Figure 2), as well as immunoglobulin deposits and complement molecules. Several pieces of laboratory evidence have revealed their essential role in the pathological changes that lead to PVR. Inflammatory cells release cytokines and mitogen factors that stimulate RPE and glial cells to transdifferentiate, proliferate, and deposit fibrous tissue, resulting in epiretinal and subretinal membrane formation [27,28,29,30,31].

The most important factors involved in this process are the platelet-derived growth factor (PDGF), the hepatocyte growth factor (HGF), the VEGF, the Epidermal Growth Factor (EGF), the granulocyte colony stimulating factor (G-CSF), the fibroblast growth factors a and b (aFGF and bFGF), the insulin-like growth factor 1 (IGF1), the transforming growth factors α and β (TGF-α and TGF-β), the tumor necrosis factor α (TNF-α), the interferons β and γ (IFN-β and INFγ), the interleukins 1, 1β, 6, 8, and 11(IL-1, IL-1β, IL-6, IL-8, and IL-11), and chemokines, such as the ligand of the chemokines CC 3, 4, and 5 (CCL3, CCL4, and CCL5) [31,32,33,34,35,36,37,38,39,40,41,42,43,44,45,46]. Growth factors, cytokines, and chemokines may be potential biomarkers for predicting the development and severity of PVR. 

Another key point to understanding PVR pathogenesis is the dysregulation and remodeling of the extracellular matrix (ECM) microenvironment. ECM composition in PVR preretinal membranes has been the subject of immunohistochemistry studies. Morino et al. [47] demonstrated the consistent presence of interstitial collagen of types I and III and the inconsistent presence of type II collagen. Non-collagenous extracellular components of preretinal membranes include glycoproteins (laminin, fibronectin, and tenascin) among whom fibronectin has pivotal importance for cellular adhesion. Fibronectin matrix assembly seems to represent an early step in pathologic ECM protein accumulation. Both plasma- and cell-derived fibronectin can be found in PVR; the first appears as a result of vascular leakage; the second is directly produced by local cells [48]. Fibronectins promote cell–cell and cell–substrate adhesion and are responsible for providing early structural integrity in preretinal membranes and for the formation of a “contractile unit” [49].

The balance between ECM production and degradation is tightly regulated, and matrix metalloproteinases (MMPs) play an important role in this context [50]. In PVR, ECM synthesis, secretion, and degeneration are stimulated not only by MMPs but also by growth factors, especially TGF-β1 [51].

PVR-derived RPE cells show an altered integrin expression profile if compared to quiescent RPE cells [52] and are likely to be better equipped to engage with fibronectin protein and thus to initiate fibronectin matrix assembly, leading to ECM accumulation [53]. A key role in the pathogenesis of PVR is played by TGF-β2, a pro-fibrotic growth factor known to be elevated in the vitreous of eyes with PVR [54]. RPE cells treated with a transforming growth factor (TGF)-β2 are induced to express fibronectin containing extra domain A (FN-EDA), and this effect is further amplified by co-treatment with a connective tissue growth factor (CTGF) [55].

Proliferation, migration, and shape change require the partial detachment of cells from their substrate. ECM in wounds contains anti-adhesive proteins, often belonging to a group of proteins known as “matricellular” proteins, which may facilitate cell detachment and hence permit cell proliferation and migration. Matricellular proteins include tenascin, thrombospondin 1 and 2 (TSP1 and TSP2), Secreted Protein Acidic and Rich in Cysteine (SPARC), and osteopontin. Tenascin, TSP1, and SPARC have been described in PVR membranes, and an association between TSP1 and RPE cells, as well as between SPARC and RPE cells, has been observed in these membranes [56].

The levels of both signal transduction proteins and early apoptosis proteins were recently studied in SRF, collected by evacuative drainage during episcleral surgery for macula-off RRDs. The study showed that these proteins are associated with different clinical features and post-surgical outcomes. A lower concentration of caspase-9 was found in the SRF of eyes that developed PVR within 6 months postoperatively, suggesting a protective role of apoptosis activation against the development of PVR. Reduced levels of pro-apoptotic proteins in retinal cells could lead to other forms of cell death, which would increase intraocular inflammation after RRD. The increase in inflammation could generate a cascade of tissue responses that generate and amplify the hostile microenvironment, where the activated RPE cells can migrate and transdifferentiate, resulting in PVR development. Another interesting finding was that the concentration of the p53 protein increased in the SRF of eyes that did not show photoreceptor damage on Optical Coherence Tomography examination. Once activated, following cellular stress, p53 induces a cell cycle arrest to allow for either repair and survival or apoptosis. This indicates that the stress following RD could induce an increase in the concentration of p53, which appears to be protective for photoreceptors, until surgery is performed. Finally, it was demonstrated that higher subretinal ERK1/2 protein concentrations are related to better visual acuities 6 months after surgery, suggesting the functional importance of this protein [57].

TGF-β is a multifunctional cytokine that regulates the differentiation, migration, apoptosis, and function of the immune cells as well as the synthesis of the extracellular matrix [58,59]. High TGF-β concentrations were found in the vitreous humors of eyes with PVR, and they were directly proportional to the extent of fibrosis. TGF-β binds to its receptors to activate multiple downstream pathways, including the Smad-dependent pathway that regulates the expression of target genes, inducing an increased expression of mesenchymal markers like alpha-actin of smooth muscle (α-SMA) and vimentin and reduced expression of epithelial markers, such as the ZO-1 [60,61,62]. Tissue fibrosis and contraction are critically dependent on the TGF-β/Smad pathway. Therefore, blocking the Smad signal effectively suppresses fibrogenesis reactions inhibiting EMT as well as the conversion from fibroblasts to myofibroblasts [63,64].

In eyes with PVR, a high expression of PDGF and its receptor in both the RPE cells and Muller cells was found. It was shown that the PDGF α receptor (PDGFR-α) is activated in PVR-affected eyes [65]. Other molecules such as VEGF, FGF, EGF, insulin, and HGF can indirectly activate PDGFR-α, promoting the persistence of the signaling responsible for cell survival, proliferation, and contraction involved in the development of PVR [66,67].

Another growth factor implicated in the development of PVR is the profibrotic TNF-α. It was shown that polymorphisms of profibrotic genes alone are not predisposed to the development of PVR, but if associated with the polymorphisms of the genes involved in apoptosis, they significantly increase the risk of PVR formation. This supports the theory that both processes play a key pathophysiological role in the development of PVR [68,69].

Cell death pathways can influence the development of intraretinal fibrosis. While some authors support the protective role of apoptosis [57], others consider it is a mechanism involved in the development of PVR [14,70,71]. Apoptosis has two main signaling cascades that lead to DNA fragmentation and cell death, which are both mediated by caspase [72,73,74,75]. Photoreceptor death induces the release of cytokines, which can attract and activate macrophages, Muller cells, astrocytes, and microglia. The activation of these cells leads to oxidative stress, which could further contribute to the cytotoxic effect on photoreceptors triggered by retinal detachment [76].

In addition to RPE cells, inflammatory cells and glial cells are also an active component of PVR [77]. The loss of neurons stimulates the hypertrophy of retinal glial cells (mainly Muller cells, but also astrocytes and microglia) responsible for retinal remodeling. These intraretinal changes can cause retinal stiffening and shortening, a phenomenon defined as intraretinal PVR. Changes in Muller cells start twenty-four hours after retinal detachment onset, and within three days, their cell bodies migrate toward the outer nuclear and outer plexiform layers, occupying the spaces left by dying photoreceptors with the extension of their processes in the subretinal space. Muller cells constitute only 20% of the retinal volume, and their hypertrophy cannot counteract the important loss of neurons induced by retinal detachment, resulting in a shortening of the retina [78,79,80,81].

In recent years, PVR physiopathology knowledge has enhanced thanks to studies regarding the role of non-coding RNAs. Different microRNAs (miRNAs) have been related both to RRD clinical features linked to progression toward PVR [82] and to PVR itself [83]. A positive association has been found between the expression of miR-21 and miR-34 and the duration of symptoms related to retinal detachment [82]. The expression of miR-143-3p, miR-224-5p, miR-361-5p, miR-452-5p, miR-486-3p, and miR891a-5p increased with the worsening of PVR grading [83].

## 4. PVR Staging

In 1983, the Retina Society Terminology Committee introduced the first PVR classification, including four degrees of severity [2]:

- Grade A or minimal PVR: vitreous haze, vitreous pigment clumps, and/or pigment clusters on inferior retina.
- Grade B or moderate PVR: wrinkling of inner retinal surface, retinal stiffness, vessels tortuosity, and/or rolled edges of the retinal breaks.
- Grade C or marked PVR: full thickness retinal folds in 1 (C-1), 2 (C-2), or 3 (C-3) quadrants.
- Grade D or massive PVR: fixed retinal folds in four quadrants with a wide (D-1), narrow (D-2), or closed funnel retinal detachment configuration, without visualization of the optic disc (D-3).

This classification has proven to have poor clinical value since the four grades provide an incomplete report of the disease severity due to the absence of differentiation between anterior and posterior, as well as preretinal and subretinal PVR. 

Therefore, in 1989, the Silicone Study Group modified the PVR classification, deleting grade D and replacing grade C with grades C-P (posterior form) and C-A (anterior form), both further subdivided according to the type of contraction and the extent of PVR expressed in clock hours [84,85].

**Figure 3:**
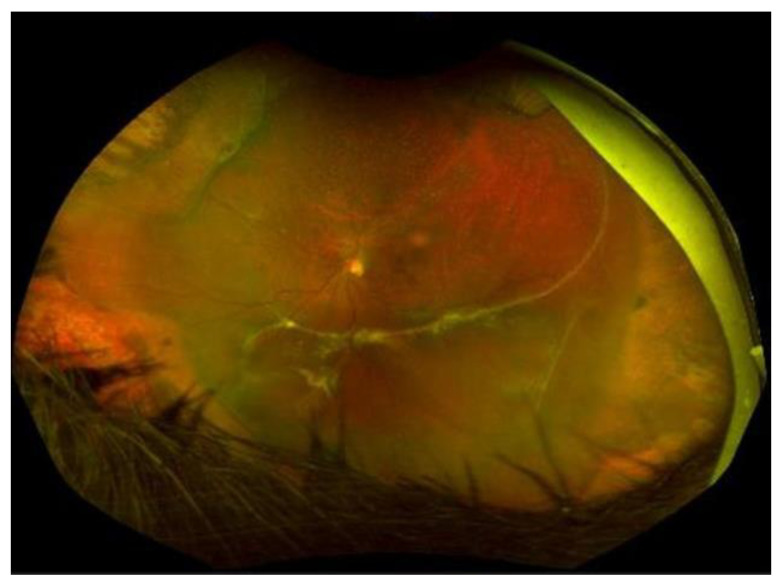
Recurrent retinal detachment due to subretinal PVR.

The latest update of the PVR classification was proposed in 1991 by the Retina Society. It improved the previous scheme retaining grades A and B unchanged and modifying grades C-P and C-A with the introduction of five types to obtain a more detailed description of the location of the proliferation and the type of contraction [86]:

- Grade C-P: Full-thickness retinal folds and/or subretinal strands (Figure 3) posterior to the equator (1–12 clock hours involvement).

Type 1: Focal contractions with formation of star folds.

Type 2: Diffuse contraction resulting from confluent star folds (optic disc may not be visible).

Type 3: Subretinal proliferation with annular or linear strands.

- Grade C-A: Full-thickness retinal folds and/or subretinal strands anterior to the equator (1–12 clock hours involvement), and anterior displacement.

Type 1: Circumferential contraction with inward traction of the retina at the posterior edge of the vitreous base.

Type 2: Anterior contraction on the retina at the vitreous base with ciliary body detachment and iris retraction.

However, due to the complexity of this classification scheme, it is rarely used in clinical practice.

A new classification should certainly consider the stage of the disease, active or quiescent, and the epiretinal, intraretinal, subretinal (shown in Figure 3), or mixed localization of PVR rather than its extent. Intraretinal changes have an enormous influence on surgical complexity and on the anatomical and functional outcomes, especially when the posterior pole is involved, but unfortunately, their clinical relevance can generally only be identified during surgery.

## 5. Therapeutic Strategies

PVR accounted for 75% of surgeries for recurrent retinal detachment [16]. To date, there is no medical therapy that has been approved for the treatment or prevention of PVR. Many pharmacological approaches have proven promising in animal model experiments, including anti-inflammatory, antiproliferative, antineoplastic, antioxidant, and antiangiogenic drugs, but none of them have passed human clinical trials [87].

Corticosteroids (CCS) were the first drugs tested for PVR treatment. Intravitreal triamcinolone acetonide showed some effectiveness in experimental animal models, but human studies revealed poor responses [88,89,90]. The addition of heparin and dexamethasone (DEX) to the infusion fluid during vitrectomy for PVR increased the rate of postoperative bleeding without any improvement in the visual outcome [91]. In a randomized prospective study including a large sample of 140 eyes affected by RRD and treated with vitrectomy, silicone oil tamponade, and intravitreal DEX implant, Banerjee et al. [34] did not observe any benefits compared with a control group in a six-month follow-up, neither in the RD recurrence rate after silicone oil removal nor in terms of visual acuity or quality of life. However, the authors reported a lower rate of postoperative macular edema in the DEX implant group than in the control group (42.7% versus 67.2%). An excessive anti-inflammatory activity that prevents retinal remodeling after RD repair could explain the poor postoperative visual outcomes in the DEX group despite the lower rate of postoperative macular edema.

Postoperative CCS therapy to prevent PVR has also been investigated. It was hypothesized that systemic CCS could reduce postoperative inflammation and counteract the increased levels of inflammatory cytokines responsible for the development of postoperative PVR. Two randomized prospective studies investigated the effectiveness of postoperative CCS and reported controversial results. Dehghan et al. [92] observed neither visual or anatomical improvement nor a reduction in postoperative complications (cystoid macular edema and PVR) using oral prednisolone 1 mg/kg/day for 10 days after surgery. Conversely, Koerner et al. [93] showed that oral prednisone at the starting dose of 100 mg/day for six days, tapered to 50 mg/day for five days, and 12.5 mg/day for four days, was effective in reducing the incidence of postoperative complications such as Grade B PVR (26.7%, 23.6%, and 19.8% in the steroid group and 41.8%, 46.9%, and 39.1% in the placebo group at one, three, and six months after surgery, respectively).

Antiproliferative and antineoplastic agents that have been studied for the prevention and treatment of PVR include 5-fluorouracil (5-FU), retinoic acid, daunorubicin, taxol, colchicine, ribozymes, vincristine, cisplatin, adriamycin, and mitomycin. 5-FU is an antimetabolite drug that inhibits the fibroblast’s proliferation and function, and it is one of the most tested drugs for PVR treatment. A large, randomized trial tested the efficacy of a combined therapy with 5-FU and low-molecular-weight heparin in RRDs with PVR, and it did not reveal any improvement in the anatomic and visual outcomes [94]. The addition of 5-FU and low-molecular-weight heparin to the infusion liquid for vitrectomy in eyes with RD relapse and PVR Grade C was recently proposed, showing significantly favorable results [95,96].

Another widely studied drug is daunorubicin (DNR), belonging to the anthracycline family, which was effective in arresting in vitro RPE cell proliferation and migration from eyes with PVR [97]. Preclinical in vivo studies on a PVR model induced by intravitreal macrophage injection showed DNR’s effectiveness in reducing the formation of PVR if injected into the vitreous cavity simultaneously with cells that are able to induce experimental PVR [98]. The study group on daunorubicin demonstrated the existence of benefits from supplementary treatment with DNR during vitrectomy in eyes with idiopathic PVR, with a significant reduction in the number of reoperations, although the rate of anatomical success after 6 months did not prove significant [99]. In recent years, several studies have tried to obtain a prolonged DNR half-life in the vitreous cavity using liposomes of porous silicon and observed the permanence of DNR therapeutic concentrations in the vitreous cavity for at least three months [100,101].

Another studied therapeutic approach is the creation of a chimeric DNA-RNA ribozyme directed against the mRNA of a cell cycle regulator protein called the Proliferating Cell Nuclear Antigen (PCNA). The hypothesis was that inhibiting PCNA, which is essential in DNA replication, would decrease the proliferation of cells involved in PVR formation after surgical procedures. The ability of the intravitreal injection of this ribozyme to prevent PVR development was tested in a rabbit PVR model with promising results in preclinical studies but failed to show anatomical and functional benefits in multicenter clinical studies [102,103].

Anti-VEGF agents were shown to be effective in inhibiting PVR formation in experimental models [104]. In a prospective randomized study of patients with PVR Grade C who underwent vitrectomy, an intravitreal injection of bevacizumab at the end of surgery resulted in better visual acuities and lower rates of PVR recurrence compared to the control group at seven months after surgery. Similar results were seen in patients with Grade B PVR [105,106].

Retinoic acid promotes the arrest of RPE cell growth in vitro. A small, randomized prospective study assessed the efficacy of oral retinoic acid in patients with PVR Grade C who underwent vitrectomy and showed a significant reduction in RD recurrence rates and an improvement in visual functions [107]. Orally administered isotretinoin (13-cis-retinoic acid) seems to be able to minimize the formation of PVR after retinal reattachment surgery, whereas there was no improvement in the success rate in established PVR [108].

Methotrexate (MTX) is a folic acid antagonist that is able to inhibit DNA/RNA synthesis in rapidly dividing cells through the blockade of dihydrofolate reductase. At low doses, MTX blocks both cell proliferation and inflammatory pathways by increasing the release of extracellular adenosine, which inhibits macrophage activation, leukocyte recruitment, and neutrophil adhesion [109]. Research about the effects of MTX on preventing or treating PVR is still debated, and the data are not resolutive. A retrospective study evaluating patients with PVR and recurrent retinal tractional detachment revealed a lower PVR incidence in patients treated with intravitreal infusion of methotrexate during vitrectomy [110]. A single intravitreal injection of MTX seemed inadequate to inhibit PVR; its prolonged proliferative cellular response must be suppressed for at least 60–90 days after primary RD. In a recent study, a series of low-dose intravitreal injections of MTX (100–200 µg/0.05 mL) for 4–5 weeks proved effective for the treatment of relapsing RDs caused by severe PVR [111]. Treatment with MTX also suppressed the inflammation that is normally associated with silicone oil tamponade [112]. In a comparative interventional non-randomized study, El Baha et al. [113] compared PPV and MTX adjuvant intravitreal infusion (equivalent to a single 400 µg/0.1 mL dose) versus PPV alone in 190 eyes, subdivided into three categories (no risk of PVR, high risk of PVR, and established PVR). They concluded that the usage of MTX gave superior functional outcomes in patients with no risk and patients at high risk of PVR but not in the established PVR group. The authors also reported that PPV and MTX do not seem to confer additional advantages in terms of the retinal reattachment rate if compared to PPV alone. Ullah A et al. [114] evaluated the usage of intravitreal low-dose MTX (200 µg/0.05 mL) during PVR surgery and every two weeks thereafter (mean: 5.6 doses). They found that the outcomes in terms of the retinal reattachment rate and visual acuity were comparable to those of other reports that used higher doses (400 µg/0.1 mL) with a lower incidence of corneal toxicity (4% vs. 10–42%). The study reported that MTX may also reduce the incidence of post-surgical hypotony in patients with PVR.

Other agents that have proven effective in preventing or treating experimental PVR models in vitro and in vivo by intervening in different phases of PVR pathogenesis are as follows:

- Taxol and colchicine stabilize and inhibit the formation of microtubules. They could reduce RPE migration and proliferation [115].
- Glucosamine is an aminomonosaccharide that suppresses RPE proliferation in vitro, interfering with the TGF-β signaling pathway [116].
- Hypericin and herbimycin A were effective in preclinical studies by reducing PVR development in animal models of RD [117,118].
- Alkylphosphocholine is a protein kinase C inhibitor that was shown to be effective against RPE migration and proliferation in vitro [119].
- AG1295, a specific PDGFR inhibitor, attenuated PVR development without significant side effects in rabbits [120].
- N-acetylcysteine is an antioxidant agent that showed efficacy in protecting rabbits from PVR formation by blocking the activation of PDGFR-α [121].
- Epigallocatechin gallate, resveratrol, and curcumin are polyphenols tested in vitro for their effect on RPE proliferation. Resveratrol was found to be the most effective by suppressing the Smad pathway in TGF-β2-treated ARPE-19 cells [122,123].
- Rho-kinase inhibitors can mitigate some of the changes triggered by RD, such as the destruction of the synaptic architecture, the apoptosis of photoreceptors, and the initiation of the EMT that characterizes PVR, promoting retinal cell survival and attenuating glial reactivity [124,125,126].
- Palomid 529 is an inhibitor of the Akt/mTOR pathways that regulate cell signaling, and in an experimental model of RD in rabbits, it was effective in suppressing Muller cell proliferation, glial scar formation, and photoreceptors death [127].
- Heavy chain hyaluronic acid/pentraxin 3 (HC-HA/PTX3), purified from human amniotic membrane, exerts anti-inflammatory and anti-scarring activities, and it was able to inhibit these PVR-related processes in vitro [128,129].
- Melatonin inhibited the proliferation and migration induced by EGF in human ARPE-19 cells by suppressing the AKT/mTOR-mediated Sp1/c-Jun signaling pathways [130].
- IL-10 and antibodies to TGF-beta2 and PDGF have been hypothesized to be therapeutic options since they can inhibit RPE-mediated retinal contraction [131].

Despite their positive results in preclinical models, most of these agents failed to show efficacy in human clinical trials. This may be due to inadequate PVR animal models and an incomplete understanding of its physiopathology. In addition, many patients who were treated with antiproliferative agents showed several side effects. The risks could be balanced by a clear advantage for a patient with a high risk of developing PVR.

Many of the failures of the already-mentioned trials depend on both the difficulty in identifying the range of efficacy and safety of such drugs and the need to maintain adequate therapeutic concentrations until the blood–retina breakdown has been fully repaired. The ideal therapy should therefore include slow-release devices that ensure adequate levels of the drug for the time necessary after surgery. It would also be useful to definitively identify the biomarkers that can point out the cessation of proliferative processes.

### Surgical Treatment

In addition to the surgical goal to reattach the retina as with all retinal detachments, the presence of PVR, especially in its higher grades, requires the surgeon to perform additional maneuvers that are aimed at solving the tractions generated by the epiretinal and subretinal membranes or to counteract the retinal shortening due to intraretinal fibrosis [5]. 

The first controversy concerns surgical timing. In the presence of the activity of PVR, some authors prefer to delay intervention by a few weeks to wait for the PVR proliferations to completely develop, allowing greater ease with membrane peeling and ensuring a more complete removal of the membranes [132]. Obviously, any decision to delay surgery must be balanced with the macula status and its implications for the visual prognosis. 

Another controversial topic concerns the right surgical choice for phakic eyes suffering from high-grade PVR. The opportunity to perform phaco-vitrectomy should be considered to obtain the complete removal of the anterior vitreous and membranes.

The advent of smaller-gauge transconjunctival pars plana vitrectomy (PPV) techniques and the technological advancement of surgical instruments have undoubtedly reduced postoperative inflammation, a well-known risk factor for the development of PVR [132].

The first base step is the complete removal of the posterior vitreous, followed by a scleral depression-assisted vitreous base shaving. Triamcinolone acetonide staining is useful for optimal detection of posterior hyaloid during posterior vitreous detachment (PVD) induction and to better visualize the peripheral vitreous [5].

The peeling of PVR epiretinal membranes (ERMs) is another crucial phase to obtain anatomical success [133]. Vital dyes, such as trypan blue and brilliant blue, highlight both preretinal membranes and the internal limiting membrane (ILM), making their removal easier. Some studies have shown that posterior pole ILM peeling reduces the risk of ERM recurrence and retinal re-detachment [134,135,136,137]. The benefits of posterior ILM peeling in the absence of PVR need more evidence.

Of no less importance was the advent of chandeliers for endoillumination, which makes it possible to perform bimanual techniques for membrane dissection and peeling in higher-grade PVR cases [138].

Subretinal PVR bands are often a challenge for the surgeon due to their difficult accessibility and their tractional effect, which rarely precludes retinal reattachment. The removal of subretinal bands can be performed through a small retinotomy or directly under the retina if a large retinectomy is necessary [5]. 

Intraoperative retinal flattening by perfluorocarbons, the so-called “third hand”, is of great utility in counteracting the tractional forces generated by membrane peeling.

Sometimes the retina cannot be completely reattached despite a complete removal of all the membranes due to a high level of intraretinal stiffness. Such cases can be approached through relaxing retinal incisions (retinotomy) or a partial retinectomy, paying attention to a perfect hemostasis at the edges of the incisions to avoid further inflammation from bleeding. 

The choice of the tamponade agent is a crucial topic. A multicenter randomized clinical trial compared long-acting gas with silicon oil (SiO) for the surgical management of PVR with vitrectomy, searching for differences in terms of postoperative visual acuity, retinal detachment recurrence rate, and incidence of complications. SiO and perfluoropropane (C3F8) were found to be equivalent concerning the rate of anatomical success and visual outcomes. Additionally, the study showed that both SiO and C3F8 were superior to sulfur hexafluoride (SF6) in terms of visual outcomes [139].

In PVR, scleral buckling (SB) plays a role in decreasing antero-posterior tractions and supporting the vitreous base, but the anatomic success rate with primary SB surgery for severe PVR retinal detachments ranges from 34 to 47% [132]. Storey et al. [140] compared pars plana vitrectomy (PPV) alone versus PPV and SB in patients deemed at high risk for PVR development. The study identified the patients as at high risk for PVR considering some clinical features: RD in two or more quadrants, retinal tears > 1 clock hour, preoperative PVR, or vitreous hemorrhage. Better anatomical results were obtained in the second group, but no significant difference between the two groups was found in terms of post-operative visual acuity. In younger patients, a higher rate of PVR was found after PPV for primary RRD; the SB procedure might be advisable as the initial surgical step in patients below 35 years of age [141].

Recently, Caporossi et al. [142] evaluated the efficacy of a modified vitrectomy procedure involving the coverage of exposed RPE in retinal breaks and retinotomy with human amniotic membrane (hAM) patches to prevent postoperative PVR in a series of 15 cases of retinal detachment complicated by severe preoperatory PVR, assuming that hAM can suppress the TGF-β pathway in a selected population of cells involved in the pathogenesis of PVR, including fibroblasts, macrophages, and particularly RPE cells.

However, despite the improvement in surgical strategies, PVR still remains one of the most complex challenges in vitreoretinal surgery, both due to the technical difficulties and the unpredictability of the results.

## 6. Conclusions

Undoubtedly, in recent decades, the knowledge of PVR’s physiopathology has improved thanks to biomolecular, genetic, and clinical studies. In addition, the enhancement of technology has permitted less invasive surgical approaches, contributing to postoperative inflammation reduction. However, a not insignificant percentage of eyes affected by retinal detachment still develop the highest grade of PVR, resulting in a very poor functional and anatomic prognosis. To date, clinical studies have not yet clarified the real significance of each single risk factor. Only knowledge of the individual’s susceptibility could guide the surgeon in choosing the most appropriate management to avoid or treat the PVR. Therefore, the first step in PVR prevention should be identifying high-risk patients through not only clinical but also biological predisposing factors. 

In this perspective, epigenetics with non-coding RNAs has gained increasing interest in the pathophysiology of many eye diseases. MiRNAs play a central role in PVR physiopathology [82,83], and there are promising possibilities to use them as disease biomarkers and master regulators for therapeutic strategies to stabilize or reset the cellular state of neurons and glial cells [143].

## Data Availability

Not applicable.
